# Clinical Validation and Implications of Dried Blood Spot Sampling of Carbamazepine, Valproic Acid and Phenytoin in Patients with Epilepsy

**DOI:** 10.1371/journal.pone.0108190

**Published:** 2014-09-25

**Authors:** Sing Teang Kong, Shih-Hui Lim, Wee Beng Lee, Pasikanthi Kishore Kumar, Hwee Yi Stella Wang, Yan Lam Shannon Ng, Pei Shieen Wong, Paul C. Ho

**Affiliations:** 1 Department of Pharmacy, National University of Singapore, Singapore, Singapore; 2 Department of Neurology, Singapore General Hospital, Singapore, Singapore; 3 Department of Neurology, National Neuroscience Institute, Singapore, Singapore; 4 Department of Neurology, Duke – National University of Singapore – Graduate Medical School, Singapore, Singapore; Alberta Children’s Hospital, Canada

## Abstract

To facilitate therapeutic monitoring of antiepileptic drugs (AEDs) by healthcare professionals for patients with epilepsy (PWE), we applied a GC-MS assay to measure three AEDs: carbamazepine (CBZ), phenytoin (PHT) and valproic acid (VPA) levels concurrently in one dried blood spot (DBS), and validated the DBS-measured levels to their plasma levels. 169 PWE on either mono- or polytherapy of CBZ, PHT or/and VPA were included. One DBS, containing ∼15 µL of blood, was acquired for the simultaneous measurement of the drug levels using GC-MS. Simple Deming regressions were performed to correlate the DBS levels with the plasma levels determined by the conventional immunoturbimetric assay in clinical practice. Statistical analyses of the results were done using MedCalc Version 12.6.1.0 and SPSS 21. DBS concentrations (C_dbs_) were well-correlated to the plasma concentrations (C_plasma_): r = 0.8381, 0.9305 and 0.8531 for CBZ, PHT and VPA respectively, The conversion formulas from C_dbs_ to plasma concentrations were [0.89×C_dbs_CBZ+1.00]µg/mL, [1.11×C_dbs_PHT−1.00]µg/mL and [0.92×C_dbs_VPA+12.48]µg/mL respectively. Inclusion of the red blood cells (RBC)/plasma partition ratio (K) and the individual hematocrit levels in the estimation of the theoretical C_plasma_ from C_dbs_ of PHT and VPA further improved the identity between the observed and the estimated theoretical C_plasma_. Bland-Altman plots indicated that the theoretical and observed C_plasma_ of PHT and VPA agreed well, and >93.0% of concentrations was within 95% CI (±2SD); and similar agreement (1∶1) was also found between the observed C_dbs_ and C_plasma_ of CBZ. As the C_plasma_ of CBZ, PHT and VPA can be accurately estimated from their C_dbs,_ DBS can therefore be used for drug monitoring in PWE on any of these AEDs.

## Introduction

Epilepsy is a neurological disease that requires chronic treatment with antiepileptic drugs (AEDs). To date, the most commonly used AEDs are still carbamazepine (CBZ), phenytoin (PHT) and valproic acid (VPA). These drugs have maximum efficacy and minimum toxicity when their plasma drug levels are within their therapeutic indexes. Hence, routine plasma concentration monitoring is recommended especially during dose adjustments, for compliance check and/or for adverse drug reaction investigation [1]. In current practice, monitoring of plasma AEDs is done using the immunoturbidimetric assay for each individual drug. In this assay, the drug of interest complexes with its specific antibody and becomes insoluble. The turbidity generated from the immune complexes corresponds to the drug concentration in sample and is then measured spectrophotometrically. However, for this assay, there is always a risk that the antibody could cross-react with the metabolites of the drug. This could result in overestimation of the plasma concentrations. During the course of AEDs therapy, approximately 40% to 50% of people with epilepsy (PWE) will require two or more antiepileptic drugs (AEDs) at one point of their therapy [2]–[4]. Attempts have therefore been made to monitor a few AEDs levels simultaneously [5]–[7], with the objective to reduce the workload of the hospital laboratories and the TDM cost borne by the patients.

Various biological matrices including cerebrospinal fluid, tear and saliva have been used for TDM [8]–[10]. In comparison with DBS as the matrix, the acquisition of blood spot is simple, and does not require the aid of phlebotomist. DBS entails small sampling volume (<100 µL) and can be acquired by patients or their caregivers at home. After drying, it can be mailed to the designated laboratory [11], [12]. The patients will be able to save their travelling time to the clinics for submitting their TDM samples. The only caveat for DBS acquisition seems to be the patients’ acceptability for the needle-prick.

Earlier studies on concurrent monitoring of multiple AEDs from one DBS were done mostly with high performance liquid chromatography (HPLC) and included whole blood concentrations of AEDs such as carbamazepine, phenytoin, lamotrigine and barbiturates with limited clinical validation [13], [14]. Recently, a group in North Ireland published a detailed HPLC ultraviolet method for concurrent determination of carbamazepine (CBZ) and its active metabolite carbamazepine-10,11 epoxide (CBZE), levetiracetam (LEV), lamotrigine (LTG) and phenobarbital (PHB) in DBS of children [15]. Similarly, they did not establish the correlations between the DBS and plasma concentrations of the AEDs involved.

In our population of PWE, CBZ, sodium valproate (VPA) and phenytoin (PHT) are the most popular antiepileptic drugs (AEDs) - used either as mono or polytherapy [4]. This has prompted us to investigate the applicability of monitoring all three AEDs using only one DBS. Considering the volatile nature of VPA and previous success in quantitation of CBZ and PHT using gas chromatography mass spectrometry (GCMS) [16], GCMS was ultimately chosen as the analytical tool for simultaneous determination of these AEDs. This study was conducted with the main objective of comparing the newly developed GCMS assay with the conventional immunoturbimetric assays. The DBS-measured concentrations were validated against their corresponding plasma concentrations as determined by the immunoturbimetric assays performed in hospital laboratory. Since plasma-to-RBC drug partitioning effect in relation to the hematocrit level is known to influence the degree of correspondence between plasma and full blood concentration of AEDs, this effect was also investigated in our study.

## Methods

This study has obtained approval from the SingHealth Institutional Review Board (CIRB No. 2011/269/A). Only PWE who had routine plasma CBZ, VPA and/or PHT, blood and liver biochemistry monitoring on the day of visit were approached for written consent prior to blood sampling.

### Patient Recruitment

Assuming constant analytical standard deviations, the sample size recommended for method validation was suggested to be at least 41 per AED based on the following information: range ratio = 2, α = 5%, power = 90%, standardized slope deviation of 4 [17], [18]. PWE who were on either CBZ, VPA or/and PHT were recruited from October 2011 to August 2012 at neurology specialist clinic of a tertiary referral hospital. This study had obtained the local ethics committee approval. Only PWE who had routine plasma CBZ, PHT and/or VPA, blood and liver biochemistries monitoring on the day of visit were included. Informed consent was obtained prior to blood sampling. PWE characteristics and biochemistry results were retrieved from clinical records and hospital information system. Each PWE was interviewed for the time of his/her last AED dose taken. Drug responses were categorized in accordance to the recent International League Against Epilepsy (ILAE) recommendations [19].

### Sampling

Venous whole blood samples were collected in EDTA tubes. Two drops of blood from the withdrawn blood, ∼30 µL each, were spotted onto 903 cards (903 Neonate Blood Collection Cards, Whatman GmbH, Dassel, Germany) and dried at room temperature, 25°C for at least 3 hours. The rest of the whole blood was sent to hospital laboratory for plasma AED quantitations as per routine protocols. To maintain direct comparability with plasma levels, DBS samples were stored at −80°C until the day of analysis. The 3 AEDs were proven to be stable at −20°C and 25°C for at least 10 days at concentrations ranging from 0.5 mg/L to 100 mg/L (Table S1).

### Plasma AEDs Quantification

The routine plasma AEDs quantifications were done in the hospital laboratory using particle enhanced turbidimetric inhibition immunoassays (Beckman Coulter Inc. Unicel DxC800, USA). The imprecisions in CV% (mean level, SD) for low, medium and high concentrations based on 21 data points over a period of typically 10 days are as following: i) Carbamazepine [range 2.0–20.0 mg/L] between run = 9.2% (4.2 mg/L, 0.39), 7.6% (10.79 mg/L, 0.82), 5.9% (15.24 mg/L, 0.93) and within run = 3.9% (3.92 mg/L, 0.15), 2.7% (10.41 mg/L, 0.28, 2.8% (15.24 mg/L, 0.43) ii) Phenytoin [2.5–40 mg/L] between run = 8.0% (5.18 mg/L, 0.42), 5.8% (15.03 mg/L, 0.83), 4.5% (30.40 mg/L, 1.36) and within run = 2.1% (5.00 mg/L, 0.11), 1.5% (13.76 mg/L, 0.20), 2.9% (28.19 mg/L, 0.80) iii) Valproic acid [10.0–150.0 mg/L] between run = 7.8% (33.26 mg/L, 2.59), 7.5% (75.90 mg/L, 5.68), 7.6% (125.70 mg/L, 9.55) and within run = 2.5% (33.03 mg/L, 0.82), 1.0% (67.99 mg/L, 0.70), 3.6% (112.83 mg/L, 4.09).

### DBS Samples Processing

Quantitation was based on one 6-mm diameter DBS punch from the centre of the spot, containing approximately 15 µL of blood. AEDs extraction was performed using 500 µL of analytical grade (99%) acetonitrile (Prime Products Pte. Ltd., Singapore) and 1 molar sodium hydroxide (JT Baker, Phillipsburg, NJ, USA) at a ratio of 24∶1, v/v with 1 µg/mL 5-(p-methylphenyl)-5-phenylhydantoin (5MP) (Sigma Aldrich, St Louis, MO) as internal standard. The extraction procedure involved 1 min of vortexing and 5 min of sonication. Then, the mixture was centrifuged for 15 min at 6000 *g*. 400 µL of supernatant was transferred into 15 mL Kimble glass tube (Gerresheimer Co. Glass, Germany) for evaporation under nitrogen gas for 15 mins at 40°C. After addition of 100 µL of toluene to the dried sample, second drying phase under similar condition was carried out. Subsequently, derivatization was attained using 50 µL of N-methyl-N-trimethylsilyltrifluoroacetamide with 1% trimethylchlorosilane (Thermo Scientific Pte. Ltd., Waltham, Massachusetts, USA) incubated at an optimum 70°C for 50 min. Derivatised samples were cooled to room temperature and diluted with 50 µL of heptane before vortexing for 1 min. Finally, 80 µL of mixture was transferred into a 200 µL conical base inert glass insert placed in a 2 mL amber glass vial (Agilent Technologies, Santa Clara, California, USA).

### Gas Chromatography Mass Spectrometry settings

The analytical assay was developed and validated with a GC-MS system that comprised of GC 2010 Shimadzu GC coupled to a GCMS-QP2010 Plus quadrupole MS (Shimadzu Corporation, Nishinokyo-Kuwabara-cho, Nakagyo-ku, Kyoto, Japan). GCMSsolution (version 2.0), was utilized for data acquisition and peak area computation. DB5 ms (30 m×0.25 mm×0.25 µm) supplied by Agilent Technologies J&W, Inc. was used as the capillary column. Injector temperature was set at 250°C, while ion source at 220°C. Injection volume of 1 µL was subjected to split ratio of 1∶5 and column flow was set at 1.9 mL/min. Column temperature began at 90°C with a 0.2-min isothermal hold. Temperature was then ramped at 4 different rates: i) 10°C/min to 120°C, held for 0.5 min ii) 65°C/min to 285°C, held for 0.5 min iii) 10°C/min to 291°C, held for 0.2 min and iv) 60°C/min to 300°C for a final isothermal hold of 5 min. Selective ion monitoring (SIM) mode was used for detection of the target analytes at their respective retention times and are tabulated in Table 1.

**Table 1:** Tabulation of selective ion monitoring (SIM) attributes and the ions monitored for the respective analytes.

Analytes	Retention Time	Quantifying Ion	Qualifying Ions
Valproic Acid	3.453 min	201	129, 145
Phenytoin	7.068 min	281	165, 176, 253
Carbamazepine	7.166 min	193	194, 293
5-methylphenylhydantoin	7.245 min	267	290, 395

### Bioanalysis

Calibration and quality control standards were prepared in blood and spotted onto the 903 cards at 30 µL each. One 6-mm diameter disc was punched out from each DBS and used for analysis. The assay was validated over a range of 0.5–120 µg/mL for all three AEDs. Accuracy ranged from 100–110% and imprecision was <10%. The calculated limit of detection was 0.05 µg/mL for VPA and approximately 0.07 µg/mL for both PHT and CBZ. The recoveries of analytes were relatively high (75%–97%) and consistent (SD≤8.2%). Although the inconsistency increased to 11% at the lower limit of quantitation for CBZ, it was still within the FDA acceptable lower limit of quantification (<20%) (Table S2).

### Statistical Analysis

DBS concentrations (C_dbs_) and plasma concentrations (C_plasma_) determined by the respective methods were directly compared. Theoretical C_plasma_ was calculated using a formula which accounts for the individual hematocrit values and red blood cell-to-plasma partition (RBC/plasma) ratio [20], [21].
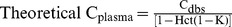
where Hct is the individual hematocrit value and K is the RBC/plasma ratio of the AEDs. To ease clinical applicability, K was fixed at literature values of 0.29 [22] and 0.43 for PHT [20] and 0.04 [23] and 0.20 [24] for VPA. As for CBZ, its theoretical plasma concentration approaches that of C_dbs_, rendering the effect of Hct on computation of theoretical C_plasma_ to be negligible. Moreover, the K for CBZ had been reported to be approximately equal to 1 [25].

Statistical analyses were done using SPSS 21 and MedCalc Version 12.6.1.0. Simple Deming regression was utilized to compare the 2 methods. The differences between methods were assessed using paired sample t-test. Bland-Altman plots were subsequently compiled using the theoretical C_plasma_ and observed C_plasma_. Outliers were confirmed using standardized score and removal was considered if the score exceeded 2.5.

## Results

### Patients

A total of 181 PWE were recruited but only 165 PWE who provided DBS were included in the final analyses. Fourteen PWE were excluded due to undetectable C_plasma_ (<2 µg/mL), and 2 PWE were excluded as outliers. Characteristics of PWE within each AED group are tabulated in Table 2. Some PWE contributed 2 AED concentrations, resulting in a total DBSs of more than 165. No significant elevation in biochemistry results was noted. The median hematocrit of the venous samples was 41.3 (range 29.8–51). The average daily doses of CBZ, PHT and VPA used in this population were 870.5±413.22 mg, 284.1±71.33 mg and 934.0±317.89 mg, respectively.

**Table 2:** Characteristics of the people with epilepsy (PWE) grouped according to the type of antiepileptic drug.

Characteristics	Valproic Acid (n = 92)	Phenytoin (n = 49)	Carbamazepine (n = 108)
Number of Eligible DBS	85	43	101
Number of Excluded DBS	7	6	7
Number of Subjects	84	41	100
Male	48 (57.1%)	22 (53.7%)	48 (48%)
Age, Median (Range)	44.3 (19–78)	50.6 (18–72)	42.9 (20–78)
Ethnic, No. Subjects (%)			
Chinese	74 (88.1%)	37 (90.2%)	89 (89%)
Malay	5 (6.0%)	3 (7.3%)	5 (5%)
Indian	3 (3.6%)	nil	6 (6%)
Others	2 (2.3%)	1 (2.4%)	nil
Concurrent medications, No. Subjects (%)			
None	7 (8.3%)	22 (53.7%)	22 (22%)
Valproic Acid	-	10 (24.4%)	46 (46%)
Phenytoin	10 (11.9%)	-	nil
Carbamazapine	46 (54.8%)	nil	-
Other AEDs	21 (25.0%)	9 (21.9%)	32 (32%)
Blood Chemistry, Median (range)			
Hematocrit (%)	41.6 (30.7–51)	42.7 (33.3–49.2)	41.3 (29.8–49.7)
Hemoglobin (g/dL)	13.9 (9.8–16.8)	14.0 (10.5–16.4)	13.6 (9.2–16.8)
Liver Function Test	Median (range)		
Albumin (g/L)	40 (31–46)	41 (31–47)	41 (31–46)
ALT (U/L)	20 (8–109)	24 (12–79)	19 (9–43)
AST (U/L)	22 (12–59)	22 (16–78)	21 (12–59)
GGT (U/L)	53 (9–333)	84 (27–417)	50 (21–213)
Drug Monitoring (µg/mL), Mean (standard deviation)			
Mean plasma levels	57.1 (22.35)*	9.7 (4.67)*	8.4 (2.32)
Mean DBS levels	29.2 (14.67)*	6.9 (3.92)*	8.3 (2.56)
Mean predicted plasma levels	57.1 (20.31)	9.7 (4.60)	8.4 (2.27)
Average dose (mg), Mean (standard deviation)			
	870.5 (413.22)	934.0 (317.89)	284.1 (71.33)
**ILAE Classification of Drug Response**			
Drug Responsive	28 (33.3%)	21 (51.2%)	31 (31%)
Drug Resistant	31 (36.9%)	9 (22.0%)	38 (38%)
Undefined	25 (29.8%)	11 (26.8%)	31 (31%)

Total recruited PWE were 183. Only 169 were included in the analysis. The remaining 14 subjects were excluded due to missing plasma levels from hospital laboratory system. (Note: Some recruited PWE contributed to the levels of two AEDs).

### DBS and plasma concentrations

**Figure 1:**
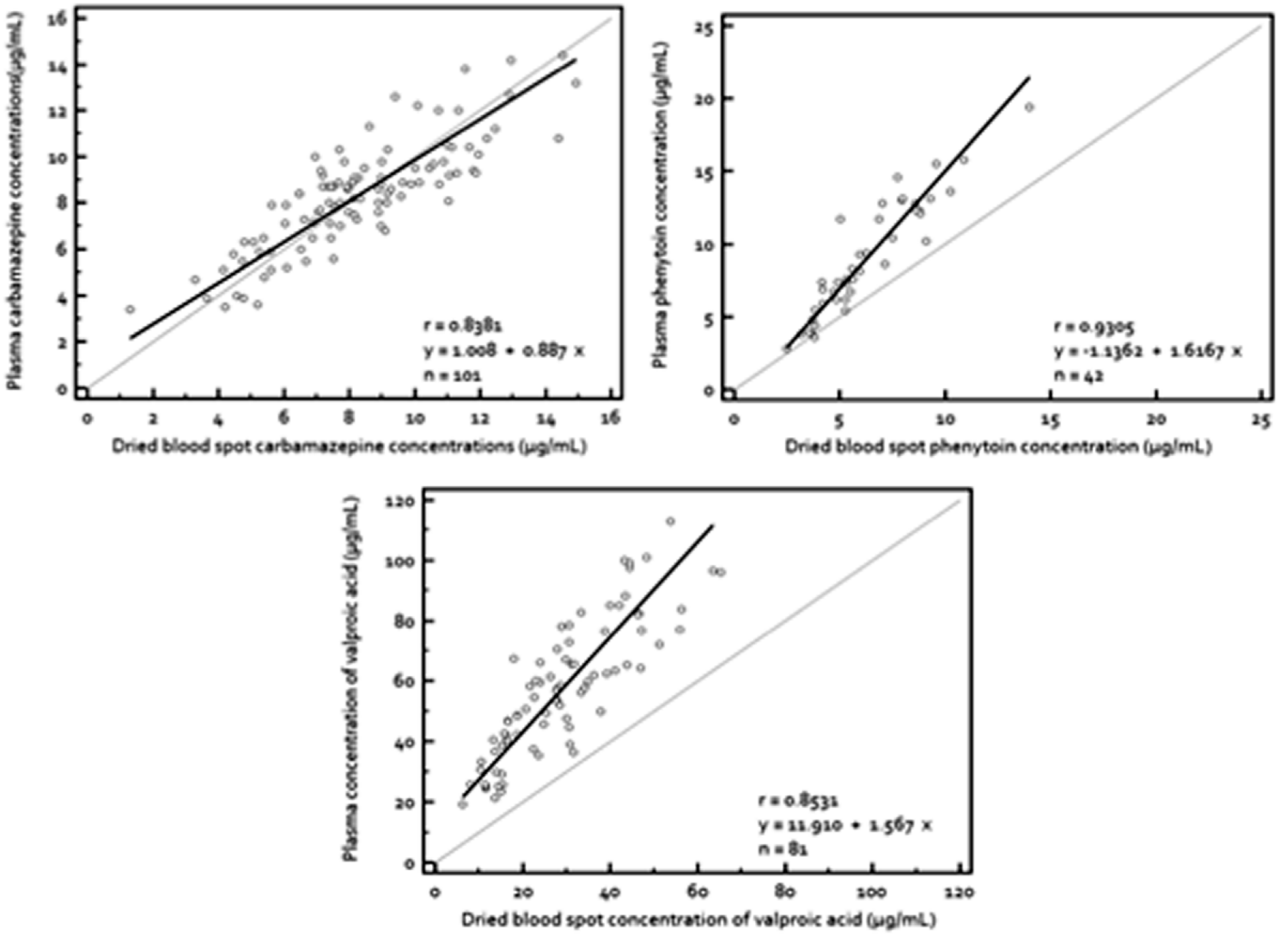
Correlation of plasma concentrations of AEDs with their corresponding dried blood spot concentrations. Plasma concentrations of (**top left**) carbamazepine (**top right**) phenytoin and (**bottom**) valproic acid regressed against their dried blood spot concentrations using Deming regression. The broken line is the line of unity while the continuous line is the line of regression. The (**top left**) slope is 0.84 (95% CI, 0.76 to 1.00) and the intercept is 1.00 (95% CI, 0.04 to 1.97) for carbamazepine, (**top right**) slope is 1.61 (95% CI, 1.39 to 1.84) and the intercept is −1.14 (95% CI, −2.40 to 0.12) for phenytoin and (**bottom**) slope is 1.57 (95% CI, 1.33 to 1.81) and the intercept is 11.91 (95% CI, 5.73 to 18.09) for valproic acid.

**Figure 1** illustrates the relationships between the C_dbs_ and C_plasma_ for the three AEDs. Good correlations were demonstrated for all three AEDs, with correlation coefficients of *r* = 0.8381, 0.9305 and 0.8531 for CBZ, PHT and VPA, respectively. C_dbs_ and C_plasma_ of CBZ were almost identical with the slope converging with line of unity. In contrast, C_dbs_ of PHT and VPA were found consistently lower than their corresponding C_plasma_, averaging at 2.8±1.89 µg/mL (29.7±13.59%) and 28.3±12.73 µg/mL (49.5±22.3%), respectively (*p<0.005*). Moreover, 95% CI for the slopes of PHT and VPA diverge anti-clockwise from the line of unity and did not cross the value 1. This signifies that there was at least a proportional increase in correlation between C_plasma_ and C_dbs_ of the two AEDs.

**Figure 2:**
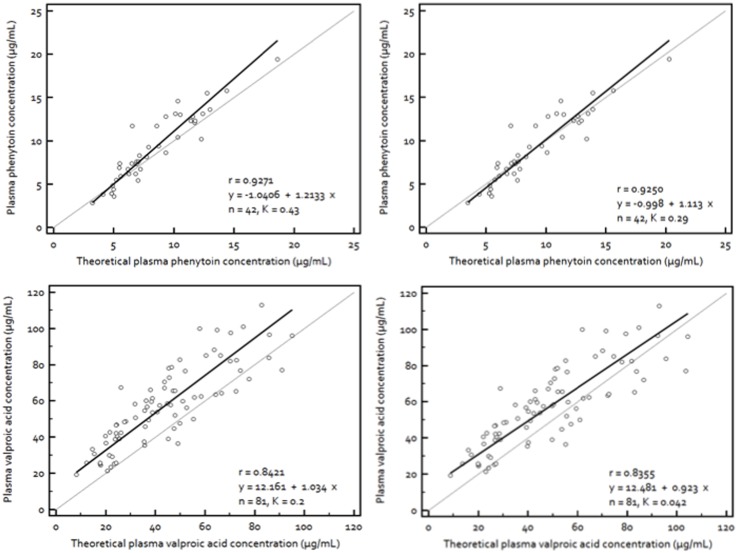
Identity of the plasma concentrations of phenytoin and valproic acid with the theoretical plasma concentrations derived from their dry blood spot concentrations. Plasma concentrations of (**top**) phenytoin and (**bottom**) valproic acid regressed against their theoretical plasma concentrations estimated from dried blood spot concentrations using Deming regression. [Theoretical plasma concentrations = Dried blood spot concentrations/1–Hct×(1–K)], where Hct is hematocrit and K is the RBC/plasma partition ratio. The broken line is the line of unity while the continuous line is the line of regression. The (**top left**) slope is 1.21 (95% CI, 1.04 to 1.38) and the intercept is −1.04 (95% CI, −2.32 to 0.24) for phenytoin with K = 0.43, (**top right**) slope is 1.11 (95% CI, 0.95 to 1.27) and the intercept is −1.00 (95% CI, −2.28 to 0.29) for phenytoin with K = 0.29, (**bottom left**) slope is 1.03 (95% CI, 0.87 to 1.20) and the intercept is 12.16 (95% CI, 5.95 to 18.37) for valproic acid with K = 0.2 and (**bottom right**) slope is 0.92 (95% CI, 0.77 to 1.07) and the intercept is 12.48 (95% CI, 6.15 to 18.81) for valproic acid with K = 0.042.

When regressed against the theoretical C_plasma_, definite improvements in fit between data points from the turbimetric assay and DBS assay were observed. The lines of regression for respective AEDs rotated closer to the line of unity and data points clustered in greater proximity along the identity line, albeit marginal decrease in correlation coefficients for PHT and VPA (**Figure 2**). The theoretical C_plasma_ of PHT was found to be comparable to its observed C_plasma_ (**Figure 2**, top). However, the regression line drawn using the theoretical C_plasma_ of PHT obtained using K of 0.29 produced a better fit with 95% CI of the slope of 1 (**Figure 2**, top left) than the one obtained using K of 0.43 (**Figure 2**, top right). As for VPA, the observed C_plasma_ was still higher than both theoretical C_plasma_ calculated using K of 0.20 (**Figure 2**, bottom left) and 0.042 (**Figure 2**, bottom right), but at the constant values of their intercepts.

**Figure 3:**
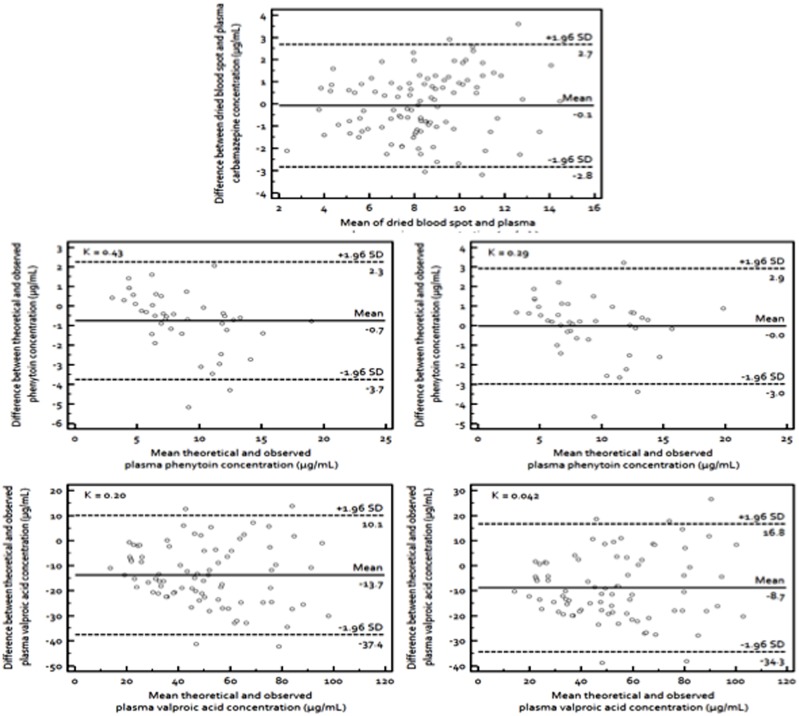
Agreement between the plasma concentrations of carbamazepine and its dried blood spot concentration; and between the plasma concentrations of phenytoin and valproic acid and the theoretical plasma concentrations derived from their dried blood spot concentrations. Bland Altman plots for plasma concentrations of (**top**) carbamazepine, (**middle left**) phenytoin, K = 0.43, (**middle right**) phenytoin, K = 0.29, (**bottom** **left**) valproic acid, K = 0.20 and (**bottom right**) valproic acid, K = 0.042. The broken lines represent the 95% CI (±1.96 SD) and the continuous line is the mean.

**Figure 3** shows the Bland-Altman plots of all three AEDs using their respective theoretical C_plasma_. The concentration used for CBZ was C_dbs_ while for PHT and VPA, the theoretical C_plasma_ was used in comparison with their observed C_plasma_. The mean difference between the concentrations from the proposed new method and conventional plasma immunoassay was −0.1 µg/mL for CBZ. For the theoretical PHT C_plasma_ calculated using K = 0.43, the mean difference was −0.7 µg/mL while with K = 0.29, the mean difference was zero. As for VPA, the differences were −13.7 and −8.1 µg/mL with K of 0.20 and 0.042, respectively. Since most of these differences fell within the acceptable limit of ±1.96 SD for all three AEDs, it is safe to assume that the theoretical C_plasma_ derived from C_dbs_ corresponded well with the observed C_plasma_. Based on the preceding discussion, the theoretical C_plasma_ of VPA estimated from K = 0.04 and PHT estimated from K = 0.29 yielded better fit with convergence to the line of unity and were therefore, recommended for clinical use.

## Discussion

In line with a presumed K of 1.06 from literatures, the RBC dilutional effect on C_dbs_ of CBZ was found to be negligible [21], [25]. Since CBZ partitions between RBC and plasma to a similar extent, the concentrations measured in whole blood were approximately the same as the plasma concentrations. This enables a direct comparison between C_dbs_ and C_plasma_ of CBZ in this study, giving a conversion factor of about 0.9.

Contrasting to CBZ, the C_dbs_ of VPA was found to be constantly lower than its C_plasma_. Similar finding were previously observed by Vermeij and Edelbroek in their study. They found a conversion factor of 1.46 from C_dbs_ to C_plasma_, which implied that the C_plasma_ of VPA was 46% higher than its C_dbs_ [13]. The lower concentrations measured from DBS could be attributed to a combination of factors. Firstly, RBC accounted for 99% of cellular space of blood and its presence may serve to dilute the VPA concentrations compared to those measured in plasma alone [26]. Secondly, for drugs with whole blood-to-plasma concentration ratio of less than (1–Hct), such as VPA, they should not partition into RBC significantly [21]. The inclusion of RBC in DBS will produce a dilution effect upon total drug concentration. It is projected that only the unbound drugs can partition into RBCs [27], [28] and that this partition is consistent at varying concentrations [29], [30]. VPA has been shown to partition into the RBC from the plasma at ratios ranging from 0.04 to 0.20 [23], [24], [29]. Since most of our patients had plasma VPA concentrations within the therapeutic ranges, and normal albumin levels, the K was presumably to be constant [29]. Hence, the relatively small amount of VPA in RBC was less likely to contribute substantial value towards the overall C_dbs_. Thirdly, due to its lipophilic nature, VPA could dissolve and detach from RBC during centrifugation of whole blood to obtain the plasma and resulted in the relatively higher concentrations observed in the plasma [31], [32]. Alternatively, despite the extraction yield of >80% (RSD<6.0%) between 1 to 250 mg/L (Table S2), the binding of VPA to 903 cards may have some effect affecting the lower C_dbs_.

After incorporating the hematocrit and K values, definitive improvement was achieved for the computation of the theoretical C_plasma_ of VPA. Yet, the theoretical C_plasma_ derived from C_dbs_ remained consistently lower than C_plasma_. The most probable explanations include the domination of RBC dilutional effect and VPA binding to 903 cards. In clinical practice, however, the potential consequence of the difference in calculated C_plasma_ from C_dbs_ may not be obvious. An example of a worst case prediction would be, for an actual C_plasma_ of 60 µg/mL, the predicted C_plasma_ could either be 48 or 72 µg/mL. Regardless of the variation in the predicted C_plasma_, physicians are likely to increase the dose if the PWE has uncontrolled seizure, hence arriving at the similar clinical decisions. Therefore, the clinical decisions premised upon C_dbs_ will be no different from the clinical decisions made using C_plasma_.

On the other hand, PHT is readily partitions into and dissociates from the RBC, with a K reported to be approximately 0.29 [22] and 0.43 in healthy patients [20]. Its whole blood-to-plasma concentration was approximately 1.33 in both studies, which was comparable to 1.14 and 1.23, before and after corrected for Hct and K, found in this study. PHT binds to hemoglobin in RBCs [33] and was shown to be released disproportionally *in vivo* [22]. For drugs with high partition ratio into RBC such as thioridazine and derivatives, the RBC concentrations tend to have better association with treatment outcomes as compared to their plasma concentrations [34]. Therefore, obtaining an overall concentration from whole blood might provide insights into uncontrolled seizure despite having optimal plasma PHT concentration.

### Effect of hematocrit and compound specific red blood cell-to-plasma ratios

In this study, we demonstrated that compound-specific RBC-to-plasma binding and individual hematocrit level could explain the difference in concentrations detected from DBS and plasma. It seems that as RBC-to-plasma partitioning approaches 1, e.g. 1.06 for CBZ, the closer the C_dbs_ is to its C_plasma_ and hematocrit level will have no dilution effect. Conversely, as RBC-to-plasma partitioning approaches 0, the dilution effect of hematocrit level becomes prominent and higher hematocrit levels lower C_dbs_. DBS does seem to equate the whole blood characteristics which were demonstrated in previous studies [21], [30]. For lipophilic drugs such as AEDs, RBC is an important and useful transporter with high capacity but low affinity to the drugs. RBC readily releases the drug it carries and equilibrates with surrounding tissues in capillary system. Although C_plasma_ is an optimal representation of tissue concentration, RBC concentration of drug may function similarly. The constant ratios of RBC/plasma water over a wide range of AEDs concentrations proved that RBC is not a saturable system [22], [29]. Therefore, RBC concentration of drug may be negligible at low concentration, but will definitely gain importance as the concentration increases.

At clinically relevant blood and liver biochemistry variations, correcting the C_dbs_ to hematocrit and K improved the theoretical prediction of C_plasma_ for PHT and VPA. Nevertheless, this study catered for the investigation of PWE with AED binding to the red blood cells and albumin in the normal ranges. It is reasonable to assume that the effects of AED binding to blood cells and albumin should not fluctuate significantly. For the former effect, the Ks were fixed at 2 values for PHT and VPA. As evidenced by the improved graphical fit, correspondence with the actual C_plasma_ was improved with the inclusion of K. Similary, a constant K value has also been used to enhance the clinical applicability and harmonize the analytical process of PHT and VPA in whole blood samples [22], [29],.

The PWE recruited in this study represented the typical population of PWE who required the routine therapeutic drug monitoring. However, there were no inclusion of children, critically ill patients nor subjects who have just started the AED/s treatment. Hence, the applicability of this DBS quantitation method for dose titration for these cohorts of subjects remains unknown. These subjects could have a more fluctuating Hct and/or drug levels. Their theoretical C_plasma_ would be more challenging to be determined from the corresponding C_dbs_ levels. The applicability of C_dbs_ as a surrogate to C_plasma_ and even seizure control in these subjects need to be established in the future studies.

It is noteworthy that on this study, the DBS was obtained from venous source. Theoretically, there could be some differences between capillary and venous concentrations but the differences for a majority of xenobiotics were proven not to be obvious, especially after the distribution phase [35]–[37]. Therefore, at steady state drug concentrations, the difference between capillary and venous concentrations should be negligible. Blood concentrations from either source could be used interchangeably for estimating the AEDs C_plasma_ [37]. In conclusion, the theoretical C_plasma_ can be estimated through the equations below for the respective AEDs:
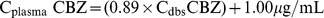


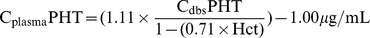


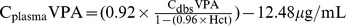
where Hct represents the individual hematocrit value, C_plasma_ CBZ, C_plasma_ PHT and C_plasma_ VPA represents the plasma concentrations of CBZ, PHT and VPA, respectively, while C_dbs_ CBZ, C_dbs_ PHT and C_dbs_ VPA represents the dried blood spot concentrations of CBZ, PHT and VPA, respectively For the theoretical C_plasma_ of PHT and VPA, the conversion factors, K = 0.29 and K = 0.04, were respectively recommended due to its proximity to the line of unity.

## Conclusion

In view of the good agreement between the theoretical C_plasma_ estimated from the C_dbs_ levels and the observed C_plasma_ for PHT and VPA, and also between the C_dbs_ and the observed C_plasma_ for CBZ, DBS is deemed suitable as an alternative matrix to the conventional plasma samples for TDM of AEDs in the adult population of PWE. Further studies that investigate the pharmacokinetic parameters such as clearance and apparent volume of distribution using the C_dbs_ levels and then correlate these concentrations to the treatment outcomes are warranted.

## Supporting Information

Table S1 **Stability of quality control samples for carbamazepine (CBZ), phenytoin (PHT) and valproic acid (VPA) under different storage conditions on Day 5 and Day 10.** Benchtop represents 25°C while Freezer represents −20°C. QC denotes quality control.
(DOCX)

Table S2 **Percentage of mean extraction recovery of analytes along with their respective residual standard deviation (RSD) at different concentrations in spiked blood.** The consistent and high recovery (>70%) of the analytes allowed for reliable quantitative studies.
(DOCX)
