# Supplementary material for: Clinical Validation and Implications of Dried Blood Spot Sampling of Carbamazepine, Valproic Acid and Phenytoin in Patients with Epilepsy
Source: PLoS One. 2014 Sep 25;9(9):e108190. doi: 10.1371/journal.pone.0108190 (PMC4177879; doi:10.1371/journal.pone.0108190)
Supplement: Table S1 — Stability of quality control samples for carbamazepine (CBZ), phenytoin (PHT) and valproic acid (VPA) under different storage conditions on Day 5 and Day 10. Benchtop represents 25°C while Freezer represents −20°C. QC denotes quality control. (DOCX) [file pone.0108190.s001.docx]

**Table S1.** Stability of quality control samples for carbamazepine (CBZ), phenytoin (PHT) and valproic acid (VPA) under different storage conditions on Day 5 and Day 10. Benchtop represents 25°C while Freezer represents -20°C. QC denotes quality control.

| **Day** | **QC concentration** | **Storage Condition** | **Mean % Stability** | | |
| --- | --- | --- | --- | --- | --- |
|  |  |  | **CBZ** | **PHT** | **VPA** |
| 5 | 1.5mg/L | Benchtop | -3.55 | 4.90 | 2.72 |
|  |  | Freezer | 0.51 | -15.67 | 1.20 |
|  | 15mg/L | Benchtop | 10.28 | -7.70 | -0.59 |
|  |  | Freezer | 9.60 | -12.01 | 2.49 |
|  | 80mg/L | Benchtop | 7.94 | 2.32 | -8.91 |
|  |  | Freezer | 10.10 | 0.02 | -5.82 |
| 10 | 1.5mg/L | Benchtop | 18.33 | 3.37 | 16.91 |
|  |  | Freezer | 3.90 | -12.84 | 9.27 |
|  | 15mg/L | Benchtop | 11.03 | 6.39 | 11.91 |
|  |  | Freezer | 4.52 | -9.81 | 6.93 |
|  | 80mg/L | Benchtop | 0.23 | -2.14 | 0.31 |
|  |  | Freezer | 2.02 | -6.97 | -1.28 |
