# Supplementary material for: Clinical Validation and Implications of Dried Blood Spot Sampling of Carbamazepine, Valproic Acid and Phenytoin in Patients with Epilepsy
Source: PLoS One. 2014 Sep 25;9(9):e108190. doi: 10.1371/journal.pone.0108190 (PMC4177879; doi:10.1371/journal.pone.0108190)
Supplement: Table S2 — Percentage of mean extraction recovery of analytes along with their respective residual standard deviation (RSD) at different concentrations in spiked blood. The consistent and high recovery (>70%) of the analytes allowed for reliable quantitative studies. (DOCX) [file pone.0108190.s002.docx]

**Table S2.** Percentage of mean extraction recovery of analytes along with their respective residual standard deviation (RSD) at different concentrations in spiked blood. The consistent and high recovery (>70%) of the analytes allowed for reliable quantitative studies.

| Concentration  (mg/L) | **Mean Recovery (RSD) in percentage (%)** | | | | | |
| --- | --- | --- | --- | --- | --- | --- |
|  | **Carbamazepine** | | **Phenytoin** | | **Valproic Acid** | |
| 1 | 70.28 | (11.10) | 84.22 | (8.21) | 94.67 | (4.64) |
| 10 | 89.98 | (1.73) | 96.88 | (4.76) | 92.90 | (1.13) |
| 50 | 74.62 | (3.67) | 93.60 | (3.00) | 90.34 | (5.69) |
| 250 | 89.01 | (1.57) | 80.21 | (1.02) | 80.43 | (4.10) |
